# Comparison of tris(2‐ethylhexyl) phosphate and di(2‐ethylhexyl) phosphoric acid toxicities in a rat 28‐day oral exposure study

**DOI:** 10.1002/jat.3930

**Published:** 2019-12-29

**Authors:** Guillaume Pelletier, Marc Rigden, Gen Sheng Wang, Don Caldwell, Shabana Siddique, Karen Leingartner, Ivana Kosarac, Sabit Cakmak, Cariton Kubwabo

**Affiliations:** ^1^ Hazard Identification Division, Environmental Health Science and Research Bureau Health Canada Ottawa Canada; ^2^ Scientific Service Division, Health Product and Food Branch Health Canada Ottawa Canada; ^3^ Exposure and Biomonitoring Division, Environmental Health Science and Research Bureau Health Canada Ottawa Canada; ^4^ Research Division, Tobacco Control Directorate Health Canada Ottawa Canada; ^5^ Population Studies Division, Environmental Health Science and Research Bureau Health Canada Ottawa Canada

**Keywords:** Di(2‐ethylhexyl) phosphoric acid, in vivo toxicity, organophosphate flame retardant, rat, testicular toxicity, Tris(2‐ethylhexyl) phosphate

## Abstract

Tris(2‐ethylhexyl) phosphate (TEHP, CAS no. 78‐42‐2) is a plasticizer and a flame retardant, while di(2‐ethylhexyl) phosphoric acid (DEHPA, CAS no. 298‐07‐7) is an oil additive and extraction solvent. Publicly‐available information on repeated exposure to these two related organophosphate compounds is fragmentary. Hence, adult male and female Fischer rats were exposed to TEHP (300, 1000 and 3000 mg/kg body weight [BW]/day) or DEHPA (20, 60 and 180 mg/kg BW/day) by gavage for 28 consecutive days, to assess and compare their toxicities. Although significantly impaired BW gains and evidence of TEHP enzymatic hydrolysis to DEHPA were observed only in males, exposures to the highest TEHP and DEHPA doses often resulted in similar alterations of hematology, serum clinical chemistry and liver enzymatic activities in both males and females. The squamous epithelial hyperplasia and hyperkeratosis observed in the non‐glandular forestomach of rats exposed to the middle and high DEHPA doses were most likely caused by the slightly corrosive nature of this chemical. Although tubular degeneration and spermatid retention were observed only in the testes of males exposed to the highest TEHP dose, numerous periodic acid‐Schiff stained crystalline inclusions were observed in testis interstitial cells at all TEHP dose levels. No‐observed‐adverse‐effect levels for TEHP and DEHPA are proposed, but the lower serum pituitary hormone levels resulting from TEHP and DEHPA exposures and the perturbations of testicular histology observed in TEHP‐treated males deserve further investigation. Improved characterization of the toxicity of flame retardants will contribute to better informed substitution choices for legacy flame retardants phased‐out over health concerns.

## Abstract 2

Tris(2‐ethylhexyl) phosphate (TEHP) is a plasticizer and a flame retardant, while the related di(2‐ethylhexyl) phosphoric acid (DEHPA) is an oil additive and extraction solvent. TEHP and DEHPA toxicities were compared in a rat 28‐day oral exposure study. Although TEHP hydrolysis to DEHPA was detected only in males, TEHP and DEHPA often had similar effects on the endpoints assessed in both males and females. The observed alterations of serum pituitary hormone levels and testis histological features in males warrant further investigation.

## 1 INTRODUCTION

Tris(2‐ethylhexyl) phosphate (TEHP) is a plasticizer and flame retardant mostly used in polyvinyl chloride and other polymers, while di(2‐ethylhexyl) phosphoric acid (DEHPA) has found applications as an additive in industrial and automotive gear oils and in metal extraction processes (Stuer‐Lauridsen et al., 2001). TEHP and DEHPA are categorized as high‐production volume chemicals in the United States, with aggregate import and production ranging from 1 to 10 million pounds in 2015 (https://chemview.epa.gov/chemview). The European Chemicals Agency reports annual tonnage bands of 1000‐10 000 tons for TEHP and 100‐1000 tons for DEHPA (http://www.echa.europa.eu).

The acute toxicity of TEHP in rat is low (LD_50_ > 37 g/kg body weight [BW]) (Smyth & Carpenter, 1948), but removing a 2‐ethylhexyl group from the phosphate core increases the acute toxicity of the resulting DEHPA by about an order of magnitude (LD_50_ = 4.9 g/kg BW) (Verschueren, 2001). Besides the occupational exposure of industrial workers to DEHPA, the general population can also be exposed to this chemical, which is present as an impurity in technical grade TEHP (Van Esch & World Health Organisation, 2000). Moreover, TEHP may also be enzymatically hydrolyzed to DEHPA in vivo, as suggested by the metabolism of other organophosphate compounds (Kluwe, Huff, Matthews, Irwin, & Haseman, 1985; Van Esch & World Health Organisation, 2000).

Although TEHP carcinogenicity was comprehensively studied in the eighties (National Toxicology Program, 1984), most of the publicly‐available in vivo toxicity data used for human health risk assessment are much older (MacFarland & Punte, 1966; Smyth & Carpenter, 1948). Information on DEHPA toxicity is also limited. The few published studies describing in vivo rodent exposures to DEHPA were limited to 4 or 5 days, did not report consumption of DEHPA‐spiked food and narrowly focused on hepatic functions (Lundgren & DePierre, 1987; Lundgren, Meijer, Birberg, Pilotti, & DePierre, 1988; Lundgren, Meijer, & DePierre, 1987).

Given the limited peer‐reviewed literature on TEHP and DEHPA, human health risk assessments may benefit from a methodical investigation of their in vivo toxicities. Consequently, adult male and female Fischer rats were exposed to TEHP or DEHPA by daily gavage for 28 consecutive days. Rat health and weight gains were monitored and urinary excretion of TEHP and DEHPA was measured. At the end of the dosing period, hematology, serum clinical chemistry, liver enzymatic activities and histopathology were assessed, allowing for a direct comparison of the effects of these related compounds on a broad range of endpoints.

## 2 MATERIALS AND METHODS

### 2.1 Materials

TEHP (cat no. 289922, lot no. MKBF6306V, 99.7% purity) and DEHPA (cat no. 237825, lot no. MKBK0176V, 99.7% purity) were purchased from Sigma‐Aldrich. Corn oil (Mazola brand, ACH foods) was obtained from a local grocery store. Unless otherwise stated, reagents were purchased from Sigma‐Aldrich.

### 2.2 Animal treatment

Experimental procedures followed the Canadian Council on Animal Care guidelines and were approved by Health Canada's Institutional Animal Care Committee before the initiation of the study (HCO‐ACC protocol no. 2014‐020). The animal exposure phase was based on OECD Test Guideline 407 (OECD, 2008). Pair‐housed Fischer rats (Charles River Laboratories) were acclimated to the facility (Health Guard^R^ cages [Research Equipment Co.] with ad libitum access to food [Harlan Tecklad 2014] and water in a room maintained at 22 ± 1°C and 50% ± 10% humidity on a 12‐hour light cycle [06:00‐18:00 hours]) for 2 weeks. Male (218 ± 13 g) and female (153 ± 5 g) rats (approximately 11 weeks old) were then randomly divided into seven treatment groups of 14 animals (seven males and seven females), housed singly and weighed daily thereafter. Based on a pilot dose range finding study, rats were administered TEHP (300, 1000 or 3000 mg/kg BW) or DEHPA (20, 60 or 180 mg/kg BW) in a constant volume of corn oil (5 mL/kg BW) by gavage for 28 consecutive days. Rats from the control group received the same weight‐adjusted volume of corn oil. On the 21st day of dosing, rats spent the night (17:00‐06:00 hours) in a metabolic cage to collect urine and feces.

Rats were fasted on the night following the 28th daily dosing and euthanized under isoflurane anesthesia the next morning. Blood withdrawn from the abdominal aorta was used for hematology and serum clinical chemistry analyses. Heart, liver, spleen, kidneys, adrenals, thymus and thyroid were weighed. In males, testes, epididymis, prostate and seminal glands were weighed, while ovaries and uterus were weighed in females. A 2 g piece of liver was excised, homogenized in 3 mL of 0.05 m Tris/0.15% KCl buffer, pH 7.4 and centrifuged at 9000 *g* for 20 minutes to obtain the S9 fraction. Sera and liver S9 fractions were stored at −80°C until use.

### 2.3 Hematology and clinical chemistry

An AcT 5 Diff hematology analyzer (Beckman Coulter) was used for the determination of the following hematological parameters: white blood cells (WBC), red blood cells, hemoglobin, hematocrit, mean corpuscular volume (MCV), mean corpuscular hemoglobin (MCH), mean corpuscular hemoglobin concentration (MCHC), platelet, mean platelet volume (MPV), neutrophils, lymphocytes, monocytes, eosinophils and basophils. An ABX Pentra 400 clinical analyzer (Horiba ABX Inc.) was used to measure total protein, albumin, creatinine, blood urea nitrogen (BUN), bilirubin, calcium, inorganic phosphate, cholesterol, triglycerides, alkaline phosphatase (ALP), alanine aminotransferase (ALT), aspartate aminotransferase (AST) and gamma glutamyl transferase (GGT) in serum. To test for vitamin B6 deficiency, ALT and AST activities were reassessed in serum samples supplemented with 160 μmol pyridoxal 5′‐phosphate/L (Jung, Mildner, Jacob, Scholz, & Precht, 1981). Liver S9 extracts were used to assess ethoxyresorufin‐*O*‐deethylase (EROD) activity following the protocol of Lubet, Nims, Mayer, Cameron, and Schechtman (1985), and benzyloxyresorufin‐*O*‐dealkylase (BROD) and pentoxyresorufin‐*O*‐dealkylase activities as described by Burke et al. (1985).

Serum acetylcholinesterase activity was determined using a standard spectrophotometric method (Ellman, Courtney, Andres, & Featherstone, 1961) with some modifications. Briefly, 30 μL of diluted serum was added in triplicate to the wells of a microtiter plate containing 250 μL of 0.1 m sodium phosphate buffer, pH 8.0 and 10 μL of 5,5′‐dithiobis‐2‐nitrobenzoic acid (0.32 mm final concentration). After a 10‐minute pre‐incubation at room temperature with agitation, 30 μL of acetylthiocholine iodide substrate (1.0 mm final concentration) was added. The plate was immediately read at 412 nm with readings every 2 minutes for 10 minutes (Spectramax M2; Molecular Devices). A standard curve of reduced glutathione was used to determine the change in absorbance per unit of ‐SH (Padilla, Lassiter, & Hunter, 1999). Testosterone and estradiol levels were assessed using ALPCO enzyme‐linked immunosorbent assay kits (cat. no. 55‐TESMS‐E01 and 55‐ESTRT‐E01), gonadotropin‐releasing hormone using a BIOMATIK enzyme‐linked immunosorbent assay kit (cat. no. EKC39138), while prolactin, luteinizing hormone and follicle‐stimulating hormone were assessed by an EMD Millipore Milliplex assay (cat. no. RPTMAG‐86K‐04). All assays were performed according to the manufacturers' instructions.

### 2.4 Histopathology

All tissues (brain, pituitary gland, trachea, esophagus, thyroid, thymus, heart, pancreas, spleen, liver, stomach, duodenum, jejunum, ileum, colon, rectum, adrenal glands, kidneys, bladder, mesenteric and axillary lymph nodes, skeletal muscle, prostate, seminal vesicle, coagulating gland uterus, ovaries, cervix and vagina) except testes were fixed in 10% neutral buffered formalin solution for at least 24 hours before processing and embedding. The bone surrounding the pituitary gland (10 mm × 5 mm, removed using a motorized saw) was decalcified for 24 hours at 4°C on a rotator using Richard‐Allan Scientific Decalcifying Solution (cat. no. 2205026) from Thermo Fisher Scientific. Formalin‐fixed tissues embedded in paraffin were cut in 5‐μm thick sections. Deparaffinized sections were stained using Modified Mayer's hematoxylin (Thermo Fisher Scientific; cat. no. 72804) and Instant Eosin (Thermo Fisher Scientific; cat. no. 6765040). Testes were fixed in Modified Davidson Solution (cat. no. 64133‐50) from Electron Microscopy Sciences for 48 hours and then transferred to 10% neutral buffered formalin. Deparaffinized 5‐μm testis sections were stained using periodic acid (Sigma‐Aldrich; cat. no. 395132) and Schiff's reagents (Sigma‐Aldrich; cat. no. 3952016), followed by counterstaining with Instant Hematoxylin (Thermo Fisher Scientific; cat. no. 6765015). The tissues were assessed by pathologists using available guidelines from INHAND (International Harmonization of Nomenclature and Diagnostic Criteria for Lesions in Rats and Mice) using a subjective rating for each lesion based on a scaling system recommended by Mann et al. (2012). High‐dose and vehicle control groups were evaluated first, followed by middle‐ and low‐dose treatment groups if significant lesions were observed in the high‐dose treatment groups.

Two brain coronal sections (one at optic chiasma level and the other at mid‐cerebellum level, including medulla) were assessed as described by Kaufmann et al. (2012). One section from the caudodorsal surface of the pituitary gland, sections through the cortex and medulla of both adrenals and longitudinal sections from both thyroid lobes were evaluated according to Frith, Ward, Chandra and Losco (2000). One mid‐spleen section, one longitudinal section of the thymus and one section containing several mesenteric and axillary lymph nodes were assessed using guidelines from Haley et al. (2005). INHAND guidelines were used for the remaining tissues. The lesions were graded using a scale from 0 to 5, where 0 is absent; 1 is minimal (assigned for background lesion); 2 is mild; 3 is moderate; 4 is marked; and 5 is severe. Hepatocyte necrosis, fatty change, and peribiliary infiltration of mononuclear cells were graded in a section of the left lateral lobe of the liver (Thoolen et al., 2010). One longitudinal bladder section and one transverse and longitudinal section (at the level of papilla and pelvis) of the kidney (Frazier et al., 2012), one longitudinal heart section through ventricle and atria (Jokinen et al., 2011), one transverse section of the trachea at the level of the thyroid, one section of the left lung lobe (Renne et al., 2009), one transverse section of the esophagus at the level of the thyroid, two stomach sections (one from cardiac region through pyloric sphincter and the other across the limiting ridge with forestomach and the fundic part of the glandular stomach), one section from the splenic part of the pancreas and one transverse section of the duodenum (at pancreas level), jejunum and ileum (1 cm proximal to the colon), and large intestine (2 cm proximal to the anus) (Nolte et al., 2016) were assessed.

Ovaries (one section per ovary), transverse sections of the uterine horn at the middle region from both sides and longitudinal sections of the cervix and vagina were evaluated using guidelines drafted by Dixon et al. (2014). Ovaries, cervix and vagina were staged individually (Li & Davis, 2007) and then an overall assessment of the estrous cycle phase (proestrus, estrus, metestrus and diestrus) was derived for each animal from combined morphological characteristics in the ovaries, cervix and vagina. One testis transverse section and one longitudinal section close to rete testis were evaluated based on the guidelines drafted by INHAND with stage awareness (Creasy et al., 2012). Retained spermatids, apoptosis of seminiferous epithelium, vacuolation of tubules and degeneration/atrophy of tubular epithelium were assessed. Tubular degeneration was graded from 1 to 5, where 1 means up to 5%; 2 between 5% and 25%; 3 between 25% and 50%; 4 between 50% and 75%; and 5 was >75% of tubules affected. Presence of step 19 spermatids in luminal surface of stage IX, X and XI tubules or in the basal Sertoli cell cytoplasm in stage XII tubules was considered as spermatid retention. Periodic acid‐Schiff (PAS)‐positive crystals are defined as the presence of PAS‐stained, crystalloid inclusions in the cytoplasm of interstitial cells. Epididymis sections including initial segment, caput and cauda were assessed. Exfoliation of germ cells, sperm number in the lumen of tubules, infiltration of mononuclear cells between tubules and tubule dilation were graded. One section of prostate from ventral lobe, one section of coagulating glands from each side, and one section of seminal vesicles from each side were evaluated, also based on INHAND guidelines (Creasy et al., 2012).

### 2.5 Urine sample analyses

Creatinine and total protein concentrations in overnight urine samples were measured using an ABX Pentra 400 clinical analyzer. The protocol for the quantification of TEHP and DEHPA was derived from a previously developed analytical method (Kosarac, Kubwabo, & Foster, 2016). TEHP‐d_51_ and DEHPA‐d_34_ standards were purchased from Toronto Research Chemicals. Acetonitrile, methanol and high‐performance liquid chromatography‐grade water were purchased from EMD Chemicals, Inc. Quality control normal human urine (pool of 10 individuals) was obtained from Lee Bio Solutions.

Briefly, urine samples were thawed and aliquots of 1 mL were diluted with 4% phosphoric acid (1:1). Samples were then mixed and spiked with 10 μL of 0.5 ng/μL Internal Standard mix (TEHP‐d_51_ and DEHPA‐d_34_). Solid phase extraction (SPE) cartridges (Oasis Wax, 60 mg) from Waters were conditioned by sequential addition of 2 mL acetonitrile, methanol and water. Samples were loaded on the cartridge at a flow rate of approximately 5 mL/min. Two milliliters of 2% formic acid were added to the sample tubes to rinse and collect any residuals of target analytes. The rinse solution was loaded on to the SPE cartridge, which was allowed to dry under vacuum for 1 minute. Target analytes were eluted with 4 mL of 4% ammonium hydroxide in methanol. Extracts were concentrated to near dryness under a gentle stream of nitrogen and then reconstituted in 100 μL of 5% methanol in water before analysis by ultraperformance liquid chromatography (UPLC)‐tandem mass spectrometry (MS/MS).

Analysis of the extracts was carried out on a Waters Acquity UPLC system coupled to a Waters Xevo TQD MS/MS operated in electrospray‐positive (TEHP) or ‐negative (DEHPA) mode. Chromatographic separation of target analytes was performed using a Waters Acquity UPLC BEH C_18_ column (1.7 μm × 2.1 mm × 50 mm) maintained at 40°C and attached to a Waters Van Guard BEH C_18_ pre‐column (1.7 μm × 2.1 mm × 5 mm). The mobile phase consisted of (A) 10 mm ammonium acetate in water and (B) methanol. The gradient programming was as follows: initial gradient 5% (B) to 90% (B) in 2.5 minutes, to 95% (B) in 1.75 minutes, hold for 4.2 minutes and equilibrate to 5% (B) for 4.08 minutes. The injection volume was 1.5 μL and the flow rate set at 0.22 mL/min. Quantifiers and qualifiers of multiple reaction monitoring transitions of the target analytes and internal standards used as well as associated collision energies are presented in Table 1. Source temperature, desolvation temperature and desolvation gas flow were set at 150°C, 350°C and 650 L/h, respectively. The extractor voltage was set at 3 V. Capillary voltages were set at 1.64 and 2.23 kV for positive and negative modes, respectively.

**Table 1:** Multiple reaction monitoring transitions (*m*/*z*) monitored and collision energies of target analytes

Compound	Transition	Collision energy (eV)	Cone voltage (V)	Ionization mode
TEHP	435.15 → 98.90*	16	18	ES+
	435.15 → 113.00	10	18	
TEHP d_51_	486.66 → 81.90*	22	28	ES+
	486.66 → 102.07	20	28	
DEHPA	321.01 → 78.88*	32	58	ES–
	321.01 → 208.89	18	58	
DEHPA d_34_	355.41 → 78.90*	30	60	ES–
	355.41 → 227.20	24	60	

DEHPA, di(2‐ethylhexyl) phosphoric acid; TEHP, tris(2‐ethylhexyl) phosphate.

* Quantifier transitions.

The method detection limit (MDL) and the limit of quantitation (LOQ) were determined according to the EPA Regulation 40 CFR part 136 (appendix B) method (http://www.ecfr.gov). The relative percentage recoveries were based on the internal standards recoveries. To minimize the matrix effect, an extracted calibration curve was prepared in a surrogate blank matrix, which contained no detectable levels of any of the target analytes. The best‐suited matrix was rat urine obtained from the control group of another study. The matrix‐matched calibration curve was linear over a concentration range of 0.5‐500 ng/mL with a coefficient of correlation (*r* ^2^) >0.998 for both TEHP (MDL 0.17 ng/mL; LOQ 0.58 ng/mL; recovery 77.3%) and DEHPA (MDL 0.19 ng/mL; LOQ 0.57 ng/mL; recovery 82.5%).

### 2.6 Statistical analyses

The normality and homoscedasticity of initial and final BWs, BW gains, organ weights, liver enzymatic activities, hematology and clinical chemistry datasets were assessed using the Shapiro‐Wilk and Brown‐Forsythe tests, respectively. Datasets that did not meet these assumptions were log‐transformed and their normality and homoscedasticity reassessed using the same tests. Datasets that satisfied these assumptions were analyzed by one‐way ANOVA followed by Dunnett's post‐hoc test, while those that did not were analyzed by one‐way ANOVA on ranks (Kruskal‐Wallis) followed by Dunn's post‐hoc test. Rat growth curves were analyzed by two‐way (treatment group and exposure day) repeated measure ANOVA. These analyses were performed in SigmaPlot 12.5 (Systat Software) and differences were considered statistically significant for *P* < .05. Urinary TEHP and DEHPA levels were compared using SAS Enterprise Guide 5.1 (SAS Institute). Q‐Q plots revealed that the dataset was not normally distributed. Data were log‐transformed and the normal distribution of the data was confirmed. A two‐factor (sex and treatment group) ANOVA using generalized linear models with the LSMEANS statement, which uses adjusted means (for other terms in the model) rather than simple arithmetic means, was then performed. The Bartlett's test for residuals revealed that the distribution of the error term was normal with a mean of zero, confirming the aptness of the model for the data. Differences from control group values and between males and females were assessed by the Tukey's studentized range (HSD) post‐hoc test and considered statistically significant for *P* < .05.

## 3 RESULTS

### 3.1 Rat bodyweight gains and relative organ weights

**Figure 1:**
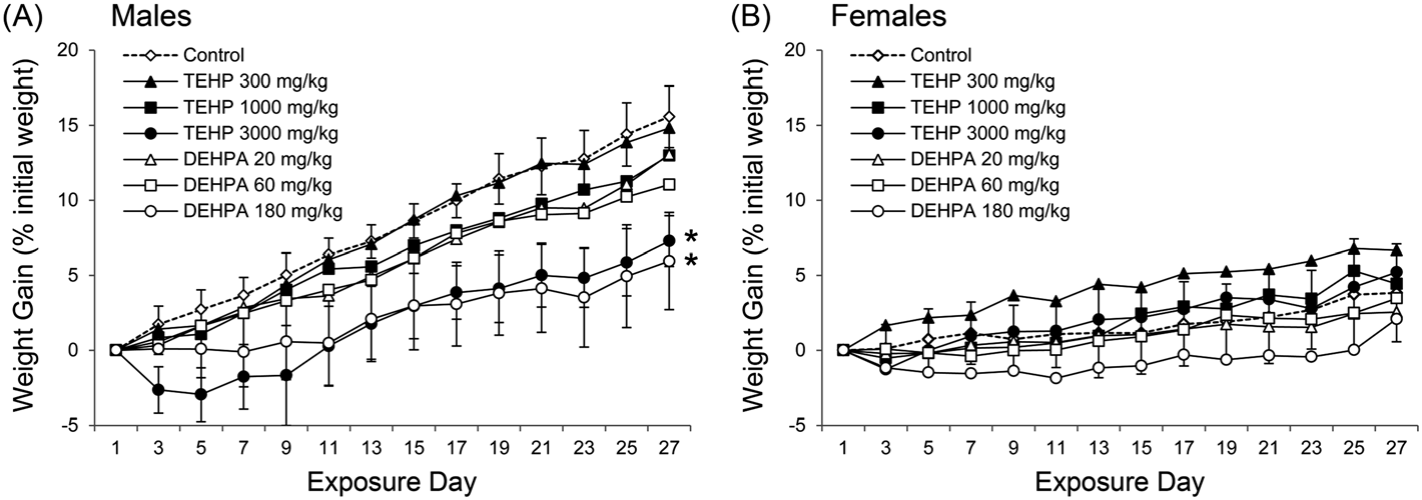
A, Male rats. B, Female rats. Growth curves of male and female rats exposed to TEHP or DEHPA by gavage for 28 consecutive days. Error bars represent standard deviations from seven animals per treatment group and are provided for the controls. *Treatment groups presenting significantly different relative bodyweight gains. DEHPA, di(2‐ethylhexyl) phosphoric acid; TEHP, tris(2‐ethylhexyl) phosphate

Rats exposed to TEHP or DEHPA by gavage for 28 consecutive days did not present any overt sign of distress or toxicity. Analysis of relative BW gains by two‐way repeated measure ANOVA identified statistically significant effects for treatment group and exposure day, and a significant interaction between treatment group and exposure day, in both males and females. However, while male daily weight gains in the high‐dose TEHP (3000 mg/kg BW/day) and DEHPA (180 mg/kg BW/day) treatment groups were significantly different from the control group, the daily weight gains of TEHP‐ and DEHPA‐treated females were not significantly different from control group females (Figure 1). The initial and final rat BWs were not significantly different across treatment groups, for both males and females (Table 2). However, when looking at the weight gains of individual rats over the 28‐day dosing period, males exposed to the high TEHP dose and to the middle and high DEHPA doses gained significantly less weight over the 28‐day dosing period than males from the control group, while female BW gains were not significantly affected. Higher relative weights for liver, spleen, kidneys and adrenal glands were also observed in males, mostly in the high‐dose TEHP and DEHPA treatment groups, while increased relative kidney weights in the high‐dose TEHP treatment group was the only statistically significant perturbation observed in females (Table 2). Relative testis, epididymis, prostate and seminal gland (in males), ovary and uterus (in females), heart, thymus and thyroid gland weights were not significantly affected (data not shown).

**Table 2:** Rat growth and relative organ weights following exposure to TEHP or DEHPA for 28 consecutive days

	Initial weight (g)	Final weight (g)	Weight gain (%)	Liver (% BW)	Spleen (% BW)	Kidneys (% BW)	Adrenals (% BW)
Male							
Control	217.7 ± 18.0	253.1 ± 20.9	16.3 ± 2.0	2.65 ± 0.08	0.207 ± 0.012	0.531 ± 0.017	0.0180 ± 0.0022
TEHP 300 mg/kg	218.9 ± 13.9	253.6 ± 12.3	16.0 ± 2.5	2.93 ± 0.20*	0.207 ± 0.009	0.524 ± 0.021	0.0182 ± 0.0016
TEHP 1000 mg/kg	219.1 ± 13.6	249.1 ± 21.2	13.6 ± 3.9	2.98 ± 0.26*	0.215 ± 0.009	0.538 ± 0.026	0.0191 ± 0.0012
TEHP 3000 mg/kg	212.7 ± 10.3	230.3 ± 11.2	8.3 ± 1.4*	2.62 ± 0.22	0.235 ± 0.012*	0.587 ± 0.044*	0.0194 ± 0.0017
DEHPA 20 mg/kg	217.7 ± 10.6	246.9 ± 11.7	13.5 ± 3.4	2.69 ± 0.12	0.214 ± 0.006	0.539 ± 0.015	0.0191 ± 0.0015
DEHPA 60 mg/kg	220.6 ± 13.4	246.5 ± 12.9	11.8 ± 3.6*	2.76 ± 0.16	0.217 ± 0.015	0.531 ± 0.020	0.0194 ± 0.0013
DEHPA 180 mg/kg	222.6 ± 17.0	238.1 ± 20.5	7.0 ± 3.2*	2.99 ± 0.05*	0.211 ± 0.013	0.573 ± 0.011*	0.0238 ± 0.0016*
Female							
Control	154.1 ± 1.9	160.9 ± 4.4	4.4 ± 3.8	2.71 ± 0.25	0.266 ± 0.049	0.601 ± 0.028	0.0387 ± 0.0062
TEHP 300 mg/kg	151.8 ± 6.9	164.2 ± 7.0	8.2 ± 2.8	3.15 ± 0.47	0.261 ± 0.014	0.601 ± 0.023	0.0392 ± 0.0031
TEHP 1000 mg/kg	152.8 ± 6.5	160.1 ± 6.9	4.8 ± 3.4	3.04 ± 0.49	0.260 ± 0.018	0.601 ± 0.030	0.0405 ± 0.0043
TEHP 3000 mg/kg	153.7 ± 4.4	161.9 ± 4.6	5.3 ± 2.5	2.90 ± 0.16	0.259 ± 0.013	0.650 ± 0.020*	0.0388 ± 0.0033
DEHPA 20 mg/kg	153.1 ± 3.5	158.4 ± 4.9	3.5 ± 3.5	2.75 ± 0.29	0.258 ± 0.013	0.605 ± 0.032	0.0400 ± 0.0053
DEHPA 60 mg/kg	152.5 ± 5.7	158.6 ± 8.7	4.0 ± 3.8	2.67 ± 0.17	0.246 ± 0.015	0.580 ± 0.019	0.0415 ± 0.0025
DEHPA 180 mg/kg	153.6 ± 6.5	154.7 ± 5.0	0.8 ± 1.8	2.89 ± 0.14	0.250 ± 0.022	0.594 ± 0.022	0.0460 ± 0.0048

BW, body weight; DEHPA, di(2‐ethylhexyl) phosphoric acid; TEHP, tris(2‐ethylhexyl) phosphate.

Weight gains are expressed as percentage gain over initial bodyweights, while organ weights are expressed as percentages of bodyweights at the terminal necropsy.

Mean ± standard deviation from seven animals per treatment group is provided.

* Statistically significant differences (*P* < .05) from control group values.

### 3.2 Urinary TEHP and DEHPA excretion

Overall, creatinine and total protein concentrations measured in overnight urine samples were significantly higher in males than in females, but they were not significantly affected by exposures to TEHP or DEHPA (Table 3). Urinary TEHP and DEHPA concentrations in control male and female rats were slightly higher than those observed in different human populations (He et al., 2018; Reemtsma, Lingott, & Roegler, 2011). Urinary TEHP concentrations measured in all TEHP treatment groups were significantly higher than control group values, in both males and females. While urinary TEHP levels measured in males and females from the same treatment groups were not statistically different, sex was significantly affected DEHPA urinary excretion. Compared with males from the same treatment groups, females presented significantly higher urinary DEHPA concentrations, at all DEHPA dose levels. However, while urinary DEHPA concentrations measured in TEHP‐treated females were undistinguishable from control background values, males from the high‐dose TEHP treatment group presented significantly higher urinary DEHPA concentrations compared with males from the control group (and to females from the high‐dose TEHP treatment group).

**Table 3:** Quantification of TEHP and DEHPA in overnight urine samples on the third week of exposure

	Creatinine (mg/mL)	Total protein (g/mg creatinine)	TEHP (ng/mg creatinine)	DEHPA (ng/mg creatinine)
Male				
Control	1.55 ± 0.59	1.31 ± 0.23	0.44 ± 0.14	1.16 ± 0.47
TEHP 300 mg/kg	1.67 ± 0.50	1.55 ± 0.41	10.3 ± 13.8*	1.93 ± 1.09
TEHP 1000 mg/kg	1.59 ± 0.32	1.34 ± 0.40	9.76 ± 4.55*	2.10 ± 0.74
TEHP 3000 mg/kg	1.21 ± 0.64	1.22 ± 0.22	985 ± 710*	5.80 ± 2.04*
DEHPA 20 mg/kg	1.14 ± 0.37	1.54 ± 0.27	2.01 ± 2.10	1.67 ± 0.52
DEHPA 60 mg/kg	1.19 ± 0.46	1.66 ± 0.46	1.04 ± 0.90	2.97 ± 1.03*
DEHPA 180 mg/kg	1.13 ± 0.42	1.42 ± 0.29	3.56 ± 3.53	23.9 ± 19.0*
Female				
Control	0.84 ± 0.47†	0.290 ± 0.103†	0.58 ± 0.19	1.67 ± 0.67
TEHP 300 mg/kg	0.64 ± 0.43†	0.307 ± 0.070†	4.56 ± 2.84*	2.03 ± 0.82
TEHP 1000 mg/kg	0.70 ± 0.26†	0.338 ± 0.151†	8.62 ± 4.98*	1.52 ± 0.43
TEHP 3000 mg/kg	1.18 ± 0.37	0.365 ± 0.117†	349 ± 155*	2.16 ± 0.56†
DEHPA 20 mg/kg	0.95 ± 0.65	0.278 ± 0.104†	2.42 ± 2.33	8.26 ± 4.64* ^,^ †
DEHPA 60 mg/kg	0.79 ± 0.44	0.305 ± 0.111†	1.01 ± 0.68	34.9 ± 21.8* ^,^ †
DEHPA 180 mg/kg	0.63 ± 0.21†	0.330 ± 0.133†	4.40 ± 7.16	85.4 ± 86.7* ^,^ †

DEHPA, di(2‐ethylhexyl) phosphoric acid; TEHP, tris(2‐ethylhexyl) phosphate.

Mean ± standard deviation from seven animals per treatment group is provided.

* Analyte levels significantly different (*p* < .05) from control group values.

† Significantly different analyte levels in females, compared with males from the same treatment group (*P* < .05).

### 3.3 Liver enzymatic activities

Significantly decreased hepatic EROD enzymatic activities were observed in males from all the TEHP treatment groups, in absence of a clear dose‐response relationship (Table 4). In females, significantly decreased EROD activities were only observed in the high‐dose TEHP and DEHPA treatment groups. Significantly lower BROD activities were also noted in males from the low‐dose DEHPA treatment group.

**Table 4:** Hepatic phase I xenobiotic metabolizing enzyme activities in rats exposed to TEHP or DEHPA

	EROD (nmol/min/mg protein)	BROD (nmol/min/mg protein)	PROD (nmol/min/mg protein)
Male			
Control	88.0 ± 8.2	95.1 ± 10.6	36.4 ± 5.3
TEHP 300 mg/kg	69.9 ± 13.4*	99.2 ± 23.2	35.6 ± 11.2
TEHP 1000 mg/kg	60.6 ± 7.7*	90.9 ± 12.6	29.3 ± 3.7
TEHP 3000 mg/kg	65.9 ± 16.8*	93.1 ± 16.2	30.8 ± 5.2
DEHPA 20 mg/kg	77.7 ± 5.1	69.2 ± 11.4*	29.5 ± 0.9
DEHPA 60 mg/kg	91.8 ± 10.8	86.5 ± 15.9	37.2 ± 4.8
DEHPA 180 mg/kg	72.9 ± 8.3	87.6 ± 13.4	33.3 ± 6.1
Female			
Control	45.6 ± 5.7	10.8 ± 15.1	9.8 ± 4.7
TEHP 300 mg/kg	40.3 ± 8.0	14.2 ± 10.3	9.3 ± 3.2
TEHP 1000 mg/kg	40.1 ± 5.5	15.4 ± 16.6	9.7 ± 5.0
TEHP 3000 mg/kg	30.0 ± 6.1*	10.4 ± 6.5	8.3 ± 2.9
DEHPA 20 mg/kg	37.2 ± 11.9	9.1 ± 10.2	8.4 ± 4.5
DEHPA 60 mg/kg	41.7 ± 10.3	9.7 ± 11.6	9.1 ± 5.0
DEHPA 180 mg/kg	25.0 ± 5.1*	14.1 ± 5.0	8.6 ± 2.4

EROD, ethoxyresorufin‐*O*‐deethylase, BROD; benzyloxyresorufin‐*O*‐dealkylase; DEHPA, di(2‐ethylhexyl) phosphoric acid; PROD; pentoxyresorufin‐*O*‐dealkylase; TEHP, tris(2‐ethylhexyl) phosphate.

Mean ± standard deviation from seven animals per treatment group is provided.

* Statistically significant difference (*P* < .05) from control values.

### 3.4 Serum clinical chemistry

**Figure 2:**
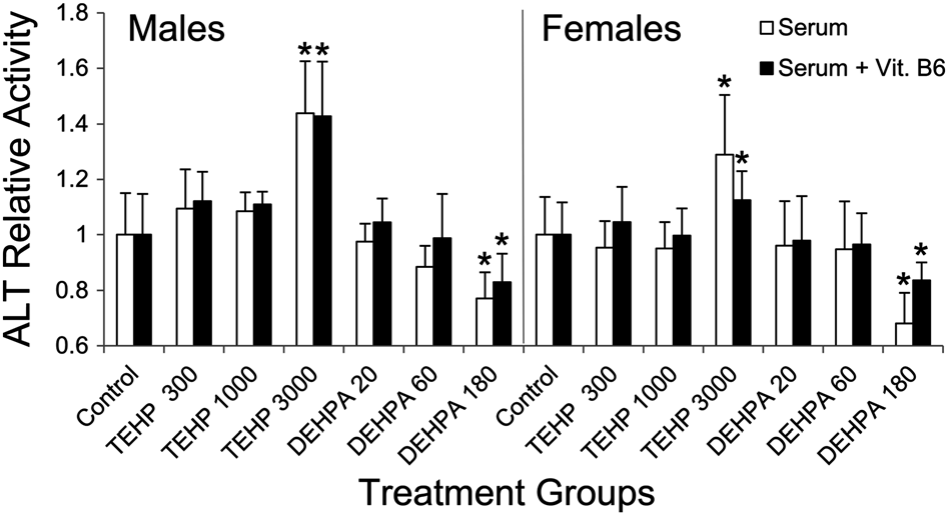
Effects of vitamin B6 supplementation on ALT activity in the serum of TEHP and DEHPA‐treated rats. Error bars represent standard deviations from seven animals per treatment group. *Statistically significant difference from control group values. ALT, alanine aminotransferase; DEHPA, di(2‐ethylhexyl) phosphoric acid; TEHP, tris(2‐ethylhexyl) phosphate

Among serum biomarkers of hepatotoxicity, ALP and GGT activities were not significantly affected in any of the treatment groups (Table 5). Serum AST activities were significantly higher in males exposed to the high TEHP dose, but lower in females exposed to the low and middle TEHP doses. Serum ALT activities were significantly increased in the high‐dose TEHP treatment group, but significantly decreased in the high‐dose DEHPA treatment group, in both males and females. Inhibition of serum ALT activity is a rather unusual observation that may have been caused by vitamin B6 deficiency (Jung et al., 1981; Ono, Ono, & Matsumata, 1995). To test this hypothesis, serum samples were supplemented with pyridoxal 5′‐phosphate (the active form of vitamin B6), and ALT activities reassessed. Vitamin B6 supplementation did not reverse the lower ALT activities observed in DEHPA‐treated males and females (Figure 2).

**Table 5:** Effects of exposure to TEHP or DEHPA on rat serum clinical chemistry

	Tot. protein (g/L)	Albumin (g/L)	Creatinine (μmol/L)	BUN (mmol/L)	Tot. bilirubin (μmol/L)	Calcium (mmol/L)	Phosphorus (mmol/L)	Cholesterol (mmol/L)	Triglycerides (mmol/L)	ALP (U/L)	ALT (U/L)	AST (U/L)	GGT (U/L)	AchE (μmol/min/mL)
Male														
Control	62.8 ± 1.9	32.3 ± 0.9	40.0 ± 3.0	5.88 ± 0.56	1.31 ± 0.34	2.55 ± 0.09	2.32 ± 0.23	1.65 ± 0.16	1.451 ± 0.259	159 ± 18	68 ± 10.2	75.8 ± 6.4	0.20 ± 0.25	0.576 ± 0.044
TEHP 300 mg/kg	65.3 ± 2.1	32.6 ± 0.9	39.7 ± 4.1	5.81 ± 0.64	1.00 ± 0.37	2.66 ± 0.09	2.50 ± 0.25	1.72 ± 0.14	1.986 ± 0.393	157 ± 18	74.4 ± 9.6	74.5 ± 5.5	0.11 ± 0.04	0.586 ± 0.031
TEHP 1000 mg/kg	65.7 ± 2.7	33.0 ± 0.9	40.0 ± 2.8	6.02 ± 0.37	1.09 ± 0.26	2.66 ± 0.11	2.42 ± 0.11	1.72 ± 0.13	1.659 ± 0.415	159 ± 19	73.8 ± 4.6	71.0 ± 3.1	0.30 ± 0.41	0.589 ± 0.043
TEHP 3000 mg/kg	66.2 ± 2.6	33.1 ± 1.1	35.3 ± 2.7*	7.44 ± 0.26*	1.19 ± 0.27	2.64 ± 0.11	2.63 ± 0.12*	1.75 ± 0.21	1.700 ± 0.550	143 ± 14	97.9 ± 12.7*	87.3 ± 5.6*	0.10 ± 0	0.576 ± 0.041
DEHPA 20 mg/kg	61.2 ± 2.3	31.4 ± 0.8	38.1 ± 1.9	5.53 ± 0.61	1.20 ± 0.32	2.57 ± 0.06	2.58 ± 0.14	1.63 ± 0.13	1.397 ± 0.340	146 ± 12	66.3 ± 4.4	73.5 ± 4.5	0.25 ± 0.34	0.564 ± 0.045
DEHPA 60 mg/kg	61.1 ± 1.0	31.7 ± 0.5	38.4 ± 3.0	5.27 ± 0.61	1.14 ± 0.28	2.59 ± 0.05	2.66 ± 0.10*	1.53 ± 0.13	1.236 ± 0.264	135 ± 13	60.2 ± 50	72.8 ± 4.0	0.14 ± 0.11	0.571 ± 0.046
DEHPA 180 mg/kg	60.3 ± 2.5	32.8 ± 1.3	36.6 ± 2.2	5.69 ± 0.44	0.96 ± 0.18	2.55 ± 0.10	2.80 ± 0.19*	1.46 ± 0.11	1.327 ± 0.358	164 ± 13	52.4 ± 6.4*	69.4 ± 3.4	0.10 ± 0	0.646 ± 0.044
Female														
Control	61.5 ± 1.4	31.5 ± 1.2	41.8 ± 2.4	6.42 ± 1.19	1.21 ± 0.19	2.51 ± 0.07	2.19 ± 0.18	2.10 ± 0.16	0.467 ± 0.124	145 ± 33	66.5 ± 9.1	83.7 ± 10	0.14 ± 0.15	1.82 ± 0.18
TEHP 300 mg/kg	63.8 ± 2.6*	32.4 ± 1.2	36.4 ± 3.0	5.88 ± 0.56	0.83 ± 0.34	2.65 ± 0.12	2.24 ± 0.27	2.10 ± 0.18	0.767 ± 0.197	110 ± 23	63.3 ± 6.4	70.7 ± 3.2*	0.10 ± 0	1.77 ± 0.18
TEHP 1000 mg/kg	66.1 ± 2.5*	32.5 ± 0.7	36.5 ± 2.9	6.59 ± 0.71	0.73 ± 0.50*	2.63 ± 0.11	2.19 ± 0.19	2.37 ± 0.16	0.973 ± 0.490*	111 ± 19	63.2 ± 6.3	70.8 ± 6.1*	0.47 ± 0.60	1.80 ± 0.33
TEHP 3000 mg/kg	66.1 ± 3.6*	32.9 ± 1.5	32.1 ± 1.4*	7.77 ± 1.47	1.31 ± 0.26	2.66 ± 0.13	2.48 ± 0.28	2.27 ± 0.34	0.767 ± 0.118*	124 ± 19	85.7 ± 14.4*	81.4 ± 7.7	0.09 ± 0.04	1.79 ± 0.18
DEHPA 20 mg/kg	63.8 ± 2.9	32.0 ± 1.0	35.1 ± 2.7	5.93 ± 0.77	1.13 ± 0.28	2.58 ± 0.09	2.39 ± 0.17	2.27 ± 0.26	0.634 ± 0.166	132 ± 27	63.8 ± 10.7	81.2 ± 10.1	0.64 ± 0.67	2.01 ± 0.24
DEHPA 60 mg/kg	62.1 ± 3.0	31.9 ± 1.4	36.0 ± 2.1	5.89 ± 0.47	1.10 ± 0.23	2.57 ± 0.10	2.42 ± 0.25	2.08 ± 0.15	0.627 ± 0.155	124 ± 32	63.0 ± 11.5	76.1 ± 8.4	0.59 ± 1.19	1.82 ± 0.27
DEHPA 180 mg/kg	60.3 ± 2.6	32.3 ± 1.8	33.3 ± 3.9*	5.50 ± 0.54	1.04 ± 0.24	2.56 ± 0.13	2.59 ± 0.24*	1.81 ± 0.28	0.547 ± 0.084	137 ± 26	45.2 ± 7.4*	74.5 ± 4.8	0.16 ± 0.14	1.51 ± 0.15

AChE, acetylcholinesterase; ALP, alkaline phosphatase; ALT, alanine aminotransferase; AST, aspartate aminotransferase; BUN, blood urea nitrogen; DEHPA, di(2‐ethylhexyl) phosphoric acid; GGT, gamma glutamyl transpeptidase; TEHP, tris(2‐ethylhexyl) phosphate.

Mean ± standard deviation from seven animals per treatment group is provided.

* Statistically significant differences (*P* < .05) from control group values.

In males, serum creatinine, BUN and phosphorus levels were significantly affected in the high‐dose TEHP treatment group, while phosphorus was significantly affected in the middle‐ and high‐dose DEHPA treatment groups (Table 5). In females, higher phosphorus levels were also observed in the high‐dose DEHPA treatment group along with lower creatinine levels in the high‐dose TEHP and DEHPA treatment groups. Additionally, total serum protein levels were significantly higher following exposure to TEHP at all doses, triglyceride levels were significantly higher in the middle‐ and high‐dose TEHP treatment groups, and lower bilirubin levels were observed in the middle‐dose TEHP treatment group.

### 3.5 Hematology

In males, significant decreases were noted for MCH at the middle TEHP and DEHPA doses, MCV at the high TEHP dose and the middle DEHPA dose and MPV at the high TEHP and DEHPA doses (Table 6). Neutrophil counts were significantly higher in the high‐dose DEHPA treatment group. In females, MPV was significantly decreased in the low‐ and high‐dose TEHP and high‐dose DEHPA treatment groups. MCH was significantly increased following exposure to high‐dose TEHP, while MCHC decreased in the high‐dose DEHPA treatment group. WBC counts were significantly higher following exposure to high‐dose TEHP, as neutrophil, lymphocyte, monocyte and eosinophil counts were all significantly higher. Neutrophils were also significantly more abundant in the middle‐ and high‐dose DEHPA treatment groups.

**Table 6:** Effects of exposure to TEHP or DEHPA on rat hematology

	WBC (×10^3^ cells/μL)	RBC (×10^6^ cells/μL)	HGB (g/dL)	HCT (%)	MCV (fL)	MCH (pg)	MCHC (g/dL)	PLT (×10^3^ cells/μL)	MPV (fL)	Neutrophils (×10^3^ cells/μL)	Lymphocytes (×10^3^ cells/μL)	Monocytes (×10^3^ cells/μL)	Eosinophils (×10^3^ cells/μL)	Basophils (×10^3^ cells/μL)
Male														
Control	3.61 ± 0.56	8.74 ± 0.28	150 ± 4	0.440 ± 0.014	50.3 ± 0.5	17.2 ± 0.2	342 ± 3	581 ± 26	6.13 ± 0.10	0.616 ± 0.142	2.68 ± 0.53	0.2486 ± 0.1044	0.0771 ± 0.0420	0.0157 ± 0.0073
TEHP 300 mg/kg	3.52 ± 0.76	8.91 ± 0.37	151 ± 6	0.443 ± 0.017	49.8 ± 0.7	17.0 ± 0.4	341 ± 3	630 ± 41	6.05 ± 0.08	0.538 ± 0.086	2.75 ± 0.66	0.1850 ± 0.0616	0.0400 ± 0.0129	0.0167 ± 0.0075
TEHP 1000 mg/kg	3.84 ± 1.09	8.88 ± 0.38	149 ± 7	0.440 ± 0.018	49.6 ± 0.5	16.8 ± 0.4*	339 ± 5	577 ± 157	6.01 ± 0.10	0.596 ± 0.127	2.97 ± 0.95	0.2000 ± 0.0635	0.0729 ± 0.0183	0.0157 ± 0.0073
TEHP 3000 mg/kg	3.80 ± 0.72	9.00 ± 0.21	152 ± 4	0.444 ± 0.011	49.1 ± 0.6*	16.8 ± 0.2	341 ± 1	631 ± 36	5.91 ± 0.10*	0.744 ± 0.125	2.67 ± 0.61	0.2629 ± 0.0468	0.1014 ± 0.0402	0.0171 ± 0.0088
DEHPA 20 mg/kg	3.33 ± 0.56	8.92 ± 0.36	150 ± 6	0.443 ± 0.018	49.9 ± 0.3	16.9 ± 0.2	339 ± 1	612 ± 26	6.13 ± 0.15	0.616 ± 0.103	2.41 ± 0.44	0.2357 ± 0.0306	0.0657 ± 0.0333	0.0157 ± 0.0090
DEHPA 60 mg/kg	3.51 ± 0.46	9.07 ± 0.31	151 ± 5	0.448 ± 0.016	49.3 ± 0.9*	16.7 ± 0.3*	337 ± 2	606 ± 52	6.10 ± 0.08	0.704 ± 0.113	2.45 ± 0.40	0.2514 ± 0.0380	0.0971 ± 0.0498	0.0157 ± 0.0073
DEHPA 180 mg/kg	3.84 ± 0.67	8.62 ± 0.43	147 ± 6	0.432 ± 0.019	50.3 ± 0.5	17.0 ± 0.2	339 ± 2	637 ± 28	5.87 ± 0.12*	0.843 ± 0.152*	2.67 ± 0.49	0.2257 ± 0.0430	0.0829 ± 0.0225	0.0186 ± 0.0064
Female														
Control	1.87 ± 0.63	7.86 ± 0.43	140 ± 7	0.406 ± 0.023	51.6 ± 0.5	17.8 ± 0.1	346 ± 2	540 ± 177	6.10 ± 0.05	0.377 ± 0.136	1.38 ± 0.50	0.0800 ± 0.0293	0.0271 ± 0.0088	0.0114 ± 0.0035
TEHP 300 mg/kg	2.03 ± 0.28	7.81 ± 0.17	140 ± 3	0.407 ± 0.008	51.8 ± 0.4	17.9 ± 0.2	344 ± 2	671 ± 46	5.81 ± 0.17*	0.366 ± 0.078	1.55 ± 0.23	0.0843 ± 0.0261	0.0271 ± 0.0116	0.0100 ± 0.0053
TEHP 1000 mg/kg	2.36 ± 0.62	7.67 ± 0.52	138 ± 9	0.397 ± 0.027	51.9 ± 0.3	18.0 ± 0.2	346 ± 2	513 ± 289	6.01 ± 0.24	0.444 ± 0.147	1.73 ± 0.45	0.0943 ± 0.0420	0.0600 ± 0.0545	0.0143 ± 0.0049
TEHP 3000 mg/kg	3.46 ± 0.74*	8.05 ± 0.14	146 ± 3	0.421 ± 0.007	52.1 ± 0.6	18.1 ± 0.3*	346 ± 4	666 ± 126	5.93 ± 0.12*	0.606 ± 0.108*	2.55 ± 0.61*	0.2014 ± 0.0479*	0.0843 ± 0.0563*	0.0200 ± 0.0093
DEHPA 20 mg/kg	2.59 ± 0.56	8.19 ± 0.18	125 ± 51	0.426 ± 0.009	52.0 ± 0	17.9 ± 0.2	345 ± 2	645 ± 36	6.03 ± 0.05	0.473 ± 0.072	1.93 ± 0.55	0.1414 ± 0.0387*	0.0300 ± 0.0160	0.0129 ± 0.0045
DEHPA 60 mg/kg	2.79 ± 0.67	8.17 ± 0.26	146 ± 5	0.424 ± 0.014	52.0 ± 0	17.9 ± 0.2	345 ± 3	670 ± 23	6.11 ± 0.10	0.616 ± 0.156*	2.00 ± 0.49	0.1243 ± 0.0424	0.0386 ± 0.0275	0.0129 ± 0.0070
DEHPA 180 mg/kg	2.83 ± 0.84	8.16 ± 0.33	144 ± 6	0.422 ± 0.018	51.9 ± 0.3	17.7 ± 0.1	342 ± 1*	671 ± 35	5.80 ± 0.14*	0.609 ± 0.149*	2.03 ± 0.70	0.1329 ± 0.0377	0.0529 ± 0.0198	0.0114 ± 0.0035

DEHPA, di(2‐ethylhexyl) phosphoric acid; HCT, hematocrit; HGB, hemoglobin; MCH, mean corpuscular hemoglobin; MCHC, mean corpuscular hemoglobin concentration; MCV, mean corpuscular volume; MPV, mean platelet volume; PLT, platelets; RBC, red blood cells; TEHP, tris(2‐ethylhexyl) phosphate; WBC, white blood cells.

Mean ± standard deviation from seven animals per treatment group is provided.

* Statistically significant differences (*P* < .05) from control group values.

### 3.6 Histopathology

Summary inspection of tissues harvested from TEHP‐ and DEHPA‐treated rats at the terminal necropsy did not reveal noticeable changes or gross lesions. Qualitative assessment of formalin‐fixed tissue sections stained with hematoxylin and eosin did not uncover treatment‐related changes in the brain, pituitary gland, trachea, esophagus, thyroid, thymus, heart, pancreas, spleen, duodenum, jejunum, ileum, colon, rectum, adrenal glands, kidney, bladder, mesenteric and axillary lymph nodes and skeletal muscle. In males, the prostate, seminal vesicle and coagulating gland presented unremarkable histological features, while in females the histological features of ovaries, uterus, cervix and vagina were not significantly affected.

**Figure 3:**
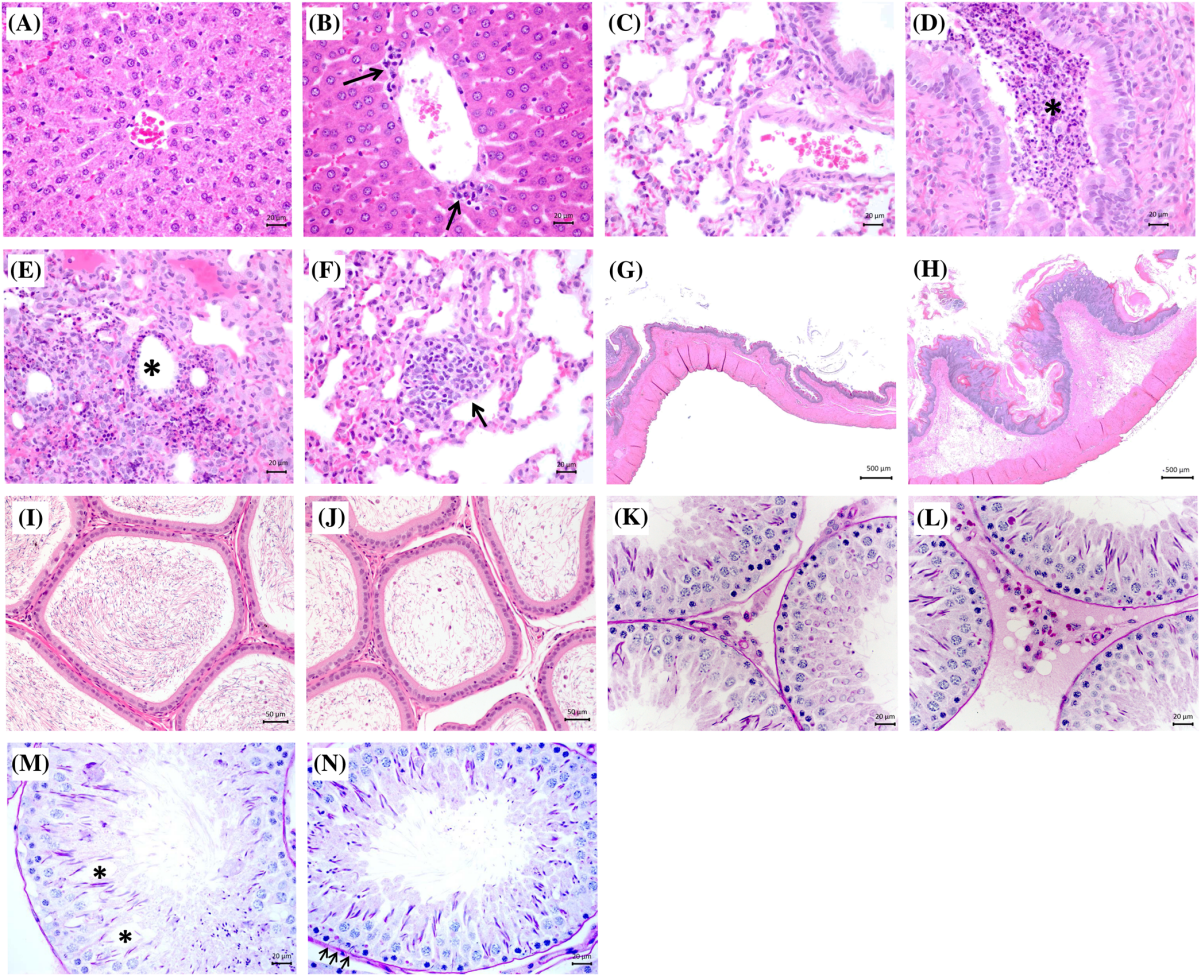
Histological perturbations observed in rats exposed to TEHP or DEHPA. A, Female liver from control group. B, Female liver from high‐dose DEHPA treatment group (arrows point to foci of minimal to moderate hepatocyte necrosis). C, Male lung from the control group. D‐F, Male lungs from the high‐dose TEHP treatment group. D, Bronchitis (*). E, Bronchiolitis (*). F, Alveolitis (arrow). G, Female non‐glandular forestomach from the control. H, Female non‐glandular forestomach from the high‐dose DEHPA treatment group where foci of squamous epithelial cell apoptosis, necrosis, hyperplasia and hyperkeratosis are visible. I, Epididymides from the control group. J, Epididymides from the high‐dose TEHP treatment group, where luminal sperm reduction and tubular exfoliation are observable. K, Testes from the control group. L‐N, Testes from the high‐dose TEHP treatment groups. L, Periodic acid‐Schiff positive crystalline inclusions in interstitial cells. M, Tubular degeneration (*). N, Spermatid retention (arrows). DEHPA, di(2‐ethylhexyl) phosphoric acid; TEHP, tris(2‐ethylhexyl) phosphate

Minimal background multifocal hepatocyte necrosis, peribiliary leukocyte infiltration and fatty changes of hepatocytes were observed in livers from all treatment groups, including control. Mild multifocal necrosis of centrilobular hepatocytes was observed in two of seven males exposed to the high TEHP dose, while only minimal necrosis was observed in males from the control group (Table 7). Mild to moderate multifocal necrosis of hepatocytes was observed in four of seven females from the high‐dose TEHP and DEHPA treatment groups, compared with two of seven in the control group (Figure 3A and 3B, Table 7). Acute multifocal bronchitis, bronchiolitis and alveolitis with mononuclear cell infiltration containing numerous neutrophils were observed in the lungs of one of seven males and two of seven females from the high‐dose TEHP treatment group (Figure 3C‐3F).

**Table 7:** Summary of the most salient histological observations in rats exposed to TEHP or DEHPA

	Liver	Stomach (squamous epithelial cell)			Testis			Epididymis		
	Multifocal centrilobular necrosis	Apoptosis	Necrosis	Hyperplasia (non‐glandular forestomach)	Spermatid retention	Tubular degeneration/atrophy	PAS‐positive crystals	Luminal sperm reduction	Exfoliation	Inflammation
Male										
Control	1.0 (0/7)	0 (0/7)	0 (0/7)	0 (0/7)	(0/7)	1.0 (0/7)	(0/7)	0 (0/7)	0 (0/7)	0 (0/7)
TEHP 300 mg/kg	1.0 (0/7)	0 (0/7)	0 (0/7)	1.1 (1/7)	(0/7)	1.1 (1/7)	(7/7)	0 (0/7)	0 (0/7)	0 (0/7)
TEHP 1000 mg/kg	1.0 (0/7)	0 (0/7)	0 (0/7)	0 (0/7)	(0/7)	1.1 (1/7)	(7/7)	0 (0/7)	0 (0/7)	0.1 (1/7)
TEHP 3000 mg/kg	1.3 (2/7)	0 (0/7)	0 (0/7)	0 (0/7)	(6/7)	2.1 (6/7)	(7/7)	3.1 (7/7)	0.9 (5/7)	0.7 (5/7)
DEHPA 20 mg/kg	1.0 (0/6)	0 (0/6)	0 (0/6)	0 (0/6)	(0/7)	1.6 (2/7)	(0/7)	0 (0/6)	0 (0/6)	0.2 (1/6)
DEHPA 60 mg/kg	1.0 (0/6)	1.5 (6/6)	1.5 (6/6)	1.5 (6/6)	(0/7)	1.3 (1/7)	(0/7)	0 (0/6)	0 (0/6)	0 (0/6)
DEHPA 180 mg/kg	1.1 (1/7)	2.0 (7/7)	2.0 (7/7)	2.0 (7/7)	(0/7)	1.3 (2/7)	(0/7)	0 (0/7)	0.1 (1/7)	0.1 (1/7)
Female										
Control	1.3 (2/7)	0 (0/7)	0 (0/7)	0 (0/7)						
TEHP 300 mg/kg	1.0 (0/7)	0 (0/7)	0 (0/7)	0 (0/7)						
TEHP 1000 mg/kg	1.0 (0/7)	0 (0/7)	0 (0/7)	0 (0/7)						
TEHP 3000 mg/kg	1.7 (4/7)	0 (0/7)	0 (0/7)	0 (0/7)						
DEHPA 20 mg/kg	1.0 (0/7)	0 (0/7)	0 (0/7)	0 (0/7)						
DEHPA 60 mg/kg	1.0 (0/7)	1.1 (6/7)	1.1 (6/7)	1.1 (6/7)						
DEHPA 180 mg/kg	1.6 (4/7)	2.0 (7/7)	2.0 (7/7)	2.0 (7/7)						

DEHPA, di(2‐ethylhexyl) phosphoric acid; PAS, periodic acid‐Schiff; TEHP, tris(2‐ethylhexyl) phosphate.

Lesions were graded as: 0 = absent, 1 = minimal (assigned for background lesion), 2 = mild, 3 = moderate, 4 = marked, or 5 = severe. PAS‐positive crystals in testes were characterized as present/absent, while tubular degeneration/atrophy was characterized as 1 = <5%, 2 = 5%‐25%, 3 = 25%‐50%, 4 = 50%‐75%, or 5 = >75% of the tubules affected. Averaged severities of the lesions are provided, along with the number of rats where the lesions were observed or present above background level (in parentheses).

Apoptosis and necrosis associated with acute non‐suppurative inflammation containing numerous neutrophils and diffuse hyperplasia and hyperkeratosis of squamous epithelial cells were observed in the non‐glandular forestomach of seven of seven males and seven of seven females exposed to the high DEHPA dose (Figure 3G and 3H, Table 7). The severity of these lesions observed in six of six males and six of seven females in the middle‐dose DEHPA treatment group decreased, and they were not observed in the control and low‐dose DEHPA treatment groups, or in TEHP‐treated rats.

Exposure to TEHP noticeably affected epididymis and testis histology. Seven of seven rats from the high‐dose TEHP treatment group presented moderate to marked epididymal luminal sperm reduction, accompanied by minimal to mild inflammation and exfoliation in five of seven rats (Figure 3I and 3J, Table 7). No such alteration was observed in control rats and minimal exfoliation and/or inflammation were observed in at most one male in the other TEHP and DEHPA treatment groups. Compared with control rat testes (Figure 3K), spermatid retention was markedly increased in six of seven rats exposed to the high TEHP dose (Figure 3N, Table 7), while no such occurrence was observed in the other treatment groups. Tubular degeneration/atrophy above background level (grades 2‐3) was observed in six of seven of the rats from the high TEHP treatment group (Figure 3M, Table 7) and in at most one or two rats in the other TEHP and DEHPA treatment groups. Numerous PAS‐stained crystalline inclusions were observed in the interstitial cells of all rats (seven of seven) from the low‐, middle‐ and high‐dose TEHP treatment groups (Figure 3L, Table 7). The size and number of these crystalline inclusions, which were observed exclusively in the testes of TEHP‐treated rats, did not appear to increase with the dose administered. These heterogeneously sized crystalloid structures frequently presented a rectangular shape with angular borders and were often found in clusters. To gain additional insights into the etiology of these testicular crystals, selected serum hormones were measured (Table 8). Significantly decreased prolactin levels were observed at all DEHPA doses and at the middle and high TEHP doses. Luteinizing hormone levels were also significantly lower in rats exposed to the middle and high DEHPA and high TEHP doses. Finally, lower levels of follicle‐stimulating hormone were observed in the high‐dose DEHPA treatment group.

**Table 8:** Serum hormone levels in male rats exposed to TEHP or DEHPA

	Estradiol (pg/mL)	Testosterone (ng/mL)	Gonadotropin‐releasing hormone (pg/mL)	Prolactin (ng/mL)	Luteinizing hormone (ng/mL)	Follicle‐stimulating hormone (ng/mL)
Control	4.76 ± 1.98	589 ± 521	4.56 ± 1.60	13.87 ± 3.47	2.63 ± 1.28	4.99 ± 0.91
TEHP 300 mg/kg	5.90 ± 2.66	248 ± 198	6.19 ± 10.18	11.71 ± 3.17	2.51 ± 1.35	4.28 ± 0.87
TEHP 1000 mg/kg	4.86 ± 2.85	401 ± 189	3.91 ± 2.04	6.04 ± 3.05*	2.76 ± 1.51	4.11 ± 0.83
TEHP 3000 mg/kg	5.42 ± 1.49	219 ± 123	2.85 ± 0.63	6.46 ± 3.01*	0.57 ± 0.58*	3.98 ± 1.01
DEHPA 20 mg/kg	6.92 ± 1.17	905 ± 1357	3.34 ± 2.24	8.55 ± 4.09*	2.21 ± 1.42	5.56 ± 1.38
DEHPA 60 mg/kg	5.98 ± 3.80	1016 ± 893	2.45 ± 1.01	4.10 ± 0.72*	0.53 ± 0.16*	3.66 ± 0.91
DEHPA 180 mg/kg	6.80 ± 3.40	398 ± 211	4.62 ± 1.45	4.72 ± 2.58*	0.51 ± 0.24*	3.08 ± 0.58*

DEHPA, di(2‐ethylhexyl) phosphoric acid; TEHP, tris(2‐ethylhexyl) phosphate.

Mean ± standard deviation from seven animals per treatment group is provided.

* Statistically significant differences (*P* < .05) from control group values.

## 4 DISCUSSION

### 4.1 Rat bodyweight gains and relative organ weights

Compared with control rats, the administration of TEHP by gavage for 28 consecutive days significantly affected growth curves and total BW gains only in males from the 3000‐mg TEHP/kg BW/day treatment group (Figure 1 and Table 2). This observation is reminiscent of a 2‐year rat carcinogenicity study where daily exposure to 2000 mg TEHP/kg BW/day impaired weight gains in males, but not in females (National Toxicology Program, 1984). Similarly, daily exposure to the high DEHPA dose significantly affected rat growth curves in males only (Figure 1), while total BW gains over the exposure period were significantly smaller than control values in males from the middle‐ and high‐dose DEHPA treatment groups (Table 2). Incidentally, higher relative organ weights were predominantly observed in treatment groups where BW gains in males were significantly affected (Table 2).

### 4.2 Urinary TEHP and DEHPA excretion

Quantification of TEHP and DEHPA in overnight urine samples yielded interesting insights on their metabolism and excretion. Tripling the TEHP dose administered from the middle‐dose to the high‐dose TEHP treatment group resulted in a 100‐fold increase in mean urinary TEHP concentrations in males (and a 40‐fold increase in females; see Table 3). This observation may hint at the saturation of sequestration, metabolism or transport mechanisms for TEHP. Compared with males from the same treatment groups, females exposed to the low, middle and high DEHPA doses presented significantly higher urinary DEHPA concentrations (Table 3). Assuming a constant DEHPA excretion rate throughout the day, a urine volume of 5.5 mL/100 g BW/day (Pass & Freeth, 1993) and a steady state by the third week of exposure, urinary DEHPA excretion in females from the high‐dose DEHPA treatment group corresponded to only 0.0015% of the daily dose administered. Hence, the higher DEHPA urinary excretion rates observed in females were simply too low to affect meaningfully the body burdens and explain the different effects of DEHPA on male and female weight gains (Figure 1 and Table 2).

While TEHP‐treated females presented urinary DEHPA concentrations similar to background control values, males from the high‐dose TEHP treatment group presented urinary DEHPA concentrations significantly higher than those measured in control males (Table 3). Based on the 99.7% purity of the TEHP batch administered (see Section 2.1), rats from the high‐dose TEHP treatment group (3000 mg/kg BW/day) may have been exposed to a maximum of 9 mg DEHPA/kg BW/day. However, males from this treatment group presented DEHPA urinary levels between those measured in males from the middle‐dose (60 mg/kg BW/day) and high‐dose (180 mg/kg BW/day) DEHPA treatment groups. This observation strongly suggests that the anticipated in vivo enzymatic hydrolysis of TEHP to DEHPA (Kluwe et al., 1985; Van Esch & World Health Organisation, 2000) occurs at a meaningful rate in males only. While it is tempting to speculate that the effects of TEHP in males may be mediated at least in part by metabolized DEHPA, it is worth noting that a few endpoints were also similarly affected by TEHP and DEHPA in females.

### 4.3 Hematology and clinical chemistry

The effects of TEHP and DEHPA on hematology were relatively limited. Most notably, exposures to TEHP and DEHPA at high doses reduced MPV in both males and females, while the high‐dose TEHP resulted in higher WBC counts in females, as neutrophil, lymphocyte monocyte and eosinophil counts were all higher (Table 6). Among serum chemistry parameters, creatinine, phosphorus and ALT stood out as they were similarly affected, either by TEHP and DEHPA exposures and/or in males and females from the same treatment group (Table 5). It is also worth noting that the activity of acetylcholinesterase, the molecular target of organophosphate pesticides, was not significantly affected following exposures to TEHP or DEHPA, supporting conclusions from an older report on TEHP toxicity (MacFarland & Punte, 1966).

### 4.4 Histopathology

Although the kidney was the only organ presenting increased relative weights in both males and females (Table 2), background lesions such as tubular basophilia, mineralization and chronic progressive nephropathy observed in the high‐dose TEHP and DEHPA treatment groups were similar in intensity and frequency to those observed in the control group. Urine creatinine and total protein levels were not significantly affected (Table 3), while serum clinical chemistry failed to provide clear clues on the perturbation of kidney functions following exposures to TEHP and DEHPA (Table 5). The higher serum phosphorus levels measured in males and females are difficult to interpret in terms of kidney function, as the organophosphate compounds administered may have interfered with this measurement. While the higher BUN levels measured in males from the high‐dose TEHP treatment group were compatible with impaired kidney functions, the lower creatinine levels measured in males and females from the same treatment group were not. The increased relative weights of adrenal glands (high‐dose DEHPA treatment group) and spleens (high‐dose TEHP treatment group) observed in males were also accompanied by unremarkable histological features.

The mild increases in squamous epithelial cell apoptosis and necrosis accompanied by diffuse mild hyperplasia and hyperkeratosis observed in the non‐glandular forestomach of all males and females exposed to the high DEHPA dose (and the minimal‐to‐mild increases observed in rats from the middle‐dose DEHPA treatment group) were not noticed in any of the control or TEHP‐treated rats (Table 7). These observations may have been caused by the corrosive nature of DEHPA and would likely have resolved upon cessation of exposure. The multifocal alveolitis, bronchiolitis and bronchitis with mononuclear cell infiltration observed in the lungs of one male and two females exposed to the high TEHP dose were most likely the consequence of accidental aspiration of the dosing solution. At the highest dose administered, TEHP accounted for more than half of the volume of the dosing solution, which may have affected the viscosity of the solution and the susceptibility to accidental lung aspiration (Damsch et al., 2011).

TEHP is a weak PXR agonist in vitro (Kojima et al., 2013). In an avian hepatocyte model, TEHP exposure was shown to induce the expression of two genes under CAR/PXR transcriptional control, while DEHPA exposure had no effect on the expression of the limited gene panel assessed in this study (Porter, Crump, Egloff, Chiu, & Kennedy, 2014). However, DEHPA presented activity in several ToxCast assays, including PXR and various peroxisome proliferator‐activated receptor subtypes (Guan, Su, Giesy, & Zhang, 2016) and rodent oral exposures to DEHPA for 4 or 5 days resulted in increased relative liver weights accompanied by the induction of hepatic enzymes (Lundgren et al., 1987; Lundgren et al., 1988; Lundgren & DePierre, 1987). In the present study, increased relative liver weights were observed only in males from the low and middle TEHP and high DEHPA doses (Table 2). Multifocal centrilobular necrosis that was slightly more pronounced than background levels was observed in a few males and females exposed to the high TEHP and DEHPA doses (Table 7). Significantly lower hepatic EROD activities were observed in males from all TEHP treatment groups, and in females from the high‐dose TEHP and DEHPA treatment groups (Table 4).

In both males and females, the serum biomarker of hepatic injury ALT was increased following exposure to high‐dose TEHP, but decreased following exposure to high‐dose DEHPA (Table 5). The lower serum ALT activities observed in kidney failure patients undergoing dialysis have been attributed to deficiencies in vitamin B6, an organophosphate molecule and coenzyme for aminotransferases (Jung et al., 1981; Ono et al., 1995). However, supplementation of rat serum with pyridoxal 5′‐phosphate did not significantly affect ALT activity, arguing against a role for vitamin B6 deficiency in the unusually low ALT activities observed in DEHPA‐treated rats (Figure 2). Overall, the relative liver weights, histology, enzymatic activities and serum biomarkers at the highest TEHP and DEHPA doses administered suggest only a mild hepatotoxicity potential for these organophosphate compounds.

### 4.5 Testicular toxicity

Testicular tubule degeneration/atrophy and spermatid retention accompanied by epididymis luminal sperm reduction and tubular exfoliation and inflammation were observed in the high‐dose TEHP treatment group (Figure 3I, 3J, 3K, 3M and 3N, Table 7). Although the TEHP‐induced testicular toxicity was not reported in a previous 2‐year carcinogenicity study (National Toxicology Program, 1984), other ethylhexyl‐containing compounds such as bis(2‐ethylhexyl)phthalate and potential metabolites (2‐ethylhexanol, 2‐ethylhexanal and 2‐ethylhexanoic acid) have been shown to affect steroidogenesis and spermatogenesis and to cause testicular toxicity in young rats (Park, Habeebu, & Klaassen, 2002; Parmar, Srivastava, & Seth, 1986; Piché et al., 2012).

Numerous PAS‐stained crystalline inclusions were also observed in testis interstitial cells from all TEHP‐treated males, at all dose levels (Figure 3L, Table 7). Contrary to human testes where Reinke crystals are frequently observed in Leydig cells (Kozina et al., 2011), the observation of such testicular crystals is a rare occurrence in rats. These crystalloid structures observed exclusively in TEHP‐treated rats may be the result of chemical‐lysosomal complex accumulation in interstitial macrophages. Alternatively, these abundant crystals may have formed in metabolically active Leydig cells, which outnumber macrophages in testis interstitium. However, such testicular crystals were not reported in a 2‐year TEHP carcinogenicity study (National Toxicology Program, 1984). This discrepancy may be explained by the different steroid synthetic activities of Leydig cells in young and senescent rats, or by different tissue fixation and staining protocols, as observed for Reinke crystals in human testicular biopsies (Mesa et al., 2015).

Reinke crystal protein constituents were tentatively identified as either nestin (Lobo et al., 2004) or calretinin and 3β‐hydroxysteroid dehydrogenase (Mesa et al., 2015; Mesa et al., 2016). Unfortunately, our first attempts to identify the protein constituents of testicular crystals from TEHP‐treated rats by immunochemistry proved unsuccessful. However, it is worth noting that while PAS strongly stains testicular crystals from TEHP‐treated rats, Reinke crystals are visible but not highlighted by this stain (Mesa et al., 2015).

Interestingly, testicular crystals can be observed in the Australian wild bush rat (*Rattus fuscipes*), a seasonal breeder that presents large seasonal fluctuations in serum testosterone, luteinizing hormone and follicle‐stimulating hormone levels (Irby, Kerr, Risbridger, & de Kretser, 1984; Kerr, Abbenhuys, & Irby, 1986). Given that TEHP can present anti‐estrogenic activities in vitro (Zhang et al., 2014), the serum levels of a few hormones were measured in males. In the absence of noticeable perturbation of pituitary gland histology, significantly lower prolactin and luteinizing hormone concentrations were observed in TEHP‐treated male rats (Table 8). However, even more pronounced decreases in prolactin, luteinizing hormone (and follicle‐stimulating hormone) levels were observed in DEHPA‐treated rats. Although these observations provide new insights in to the potential effects of TEHP and DEHPA on endocrine functions, they argue against a causative link between the observed hormonal perturbations and the PAS‐stained crystals observed in the testes of TEHP‐treated rats. Further investigations will be required to confirm the reproducibility of testicular crystal observation in TEHP‐treated rats, and to characterize better the effects of TEHP and DEHPA on endocrine functions.

## 5 CONCLUSION

Although impaired weight gains and hydrolysis of TEHP to DEHPA were observed exclusively in male rats (Figure 1, Tables 2 and 3), similar alterations of liver enzymatic activities (Table 4), serum clinical chemistry (Table 5), hematology (Table 6) and histopathology (Table 7) were often observed in both males and females. Similarities between the effects of TEHP and DEHPA were also noted. Given that the effects of TEHP on rat weight gains, organ weights and histology, hematology and clinical chemistry were mostly limited to the 3000‐mg TEHP/kg BW/day treatment group, the no‐observed‐adverse‐effect level for TEHP was estimated at 1000 mg TEHP/kg BW/day. Although the effects of DEHPA on hematology and clinical chemistry were mostly limited to the 180‐mg DEHPA/kg BW/day treatment group, epithelial cell apoptosis and necrosis accompanied by hyperplasia and hyperkeratosis were unambiguously observed in the non‐glandular forestomach of both males and females at the 60‐ and 180‐mg DEHPA/kg BW/day dose levels. Based on this salient but most likely transitory and reversible response to the mildly corrosive DEHPA, and on the impaired BW gains observed in males from the same treatment groups, the no‐observed‐adverse‐effect level for DEHPA was estimated at 20 mg DEHPA/kg BW/day. However, the interstitial cell crystals observed in the testes of all TEHP‐treated males, and the perturbations of pituitary hormones by both TEHP and DEHPA (Figure 3, Tables 7 and 8) deserve further investigation and may prove to be the most sensitive endpoints affected by these organophosphate compounds.

## CONFLICT OF INTEREST

The authors have no conflict of interest to declare.

## References

[jat3930-bib-0001] Burke, M. D. , Thompson, S. , Elcombe, C. R. , Halpert, J. , Haaparanta, T. , & Mayer, R. T. (1985). Ethoxy‐, pentoxy‐ and benzyloxyphenoxazones and homologues: A series of substrates to distinguish between different induced cytochromes P‐450. Biochemical Pharmacology, 34(18), 3337–3345. 10.1016/0006-2952(85)90355-7 3929792

[jat3930-bib-0002] Creasy, D. , Bube, A. , de Rijk, E. , Kandori, H. , Kuwahara, M. , Masson, R. , … Whitney, K. (2012). Proliferative and nonproliferative lesions of the rat and mouse male reproductive system. Toxicologic Pathology, 40(Suppl 6), 40S–121S. 10.1177/0192623312454337 22949412

[jat3930-bib-0003] Damsch, S. , Eichenbaum, G. , Tonelli, A. , Lammens, L. , Bulck, K. V. D. , Feyen, B. , … Kelley, M. (2011). Gavage‐related reflux in rats: identification, pathogenesis, and toxicological implications. Toxicologic Pathology, 39(2), 348–360. 10.1177/0192623310388431 21422261

[jat3930-bib-0004] Dixon, D. , Alison, R. , Bach, U. , Colman, K. , Foley, G. L. , Harleman, J. H. , … Yoshida, M. (2014). Nonproliferative and proliferative lesions of the rat and mouse female reproductive system. Journal of Toxicologic Pathology, 27(Suppl 3–4), 1S–107S. 10.1293/tox.27.1S 25516636 PMC4253081

[jat3930-bib-0005] Ellman, G. L. , Courtney, K. D. , Andres, V. , & Featherstone, R. M. (1961). A new and rapid colorimetric determination of acetylcholinesterase activity. Biochemical Pharmacology, 7(2), 88–95. 10.1016/0006-2952(61)90145-9 13726518

[jat3930-bib-0006] Frazier, K. S. , Seely, J. C. , Hard, G. C. , Betton, G. , Burnett, R. , Nakatsuji, S. , … Bube, A. (2012). Proliferative and nonproliferative lesions of the rat and mouse urinary system. Toxicologic Pathology, 40(4 Suppl), 14S–86S. 10.1177/0192623312438736 22637735

[jat3930-bib-0007] Frith, C. , Ward, J. , Chandra, M. , & Losco, P. (2000). Non‐proliferative lesions of the hematopoietic system in rats, HL‐1. Guides for toxicologic pathology (pp. 1–22). Washington, DC: STP/ARP/AFIP.

[jat3930-bib-0008] Guan, M. , Su, G. , Giesy, J. P. , & Zhang, X. (2016). Classification and toxicity mechanisms of novel flame retardants (NFRs) based on whole genome expression profiling. Chemosphere, 144, 2150–2157. 10.1016/j.chemosphere.2015.10.114 26588597

[jat3930-bib-0009] Haley, P. , Perry, R. , Ennulat, D. , Frame, S. , Johnson, C. , Lapointe, J. M. , & STP Immunotoxicology Working Group (2005). STP position paper: Best practice guideline for the routine pathology evaluation of the immune system. Toxicologic Pathology, 33(3), 404–407. 10.1080/01926230590934304 15805080

[jat3930-bib-0010] He, C. , Toms, L. M. L. , Thai, P. , Van den Eede, N. , Wang, X. , Li, Y. , … Covaci, A. (2018). Urinary metabolites of organophosphate esters: Concentrations and age trends in Australian children. Environment International, 111, 124–130. 10.1016/j.envint.2017.11.019 29195135

[jat3930-bib-0011] Irby, D. C. , Kerr, J. B. , Risbridger, G. P. , & de Kretser, D. M. (1984). Seasonally and experimentally induced changes in testicular function of the Australian bush rat (*Rattus fuscipes*). Journal of Reproduction and Fertility, 70(2), 657–666. 10.1530/jrf.0.0700657 6422037

[jat3930-bib-0012] Jokinen, M. P. , Lieuallen, W. G. , Boyle, M. C. , Johnson, C. L. , Malarkey, D. E. , & Nyska, A. (2011). Morphologic aspects of rodent cardiotoxicity in a retrospective evaluation of National Toxicology Program studies. Toxicologic Pathology, 39(5), 850–860. 10.1177/0192623311413788 21747121

[jat3930-bib-0013] Jung, K. , Mildner, D. , Jacob, B. , Scholz, D. , & Precht, K. (1981). On the pyridoxal‐5′‐phosphate stimulation of aspartate aminotransferase and alanine aminotransferase in serum and erythrocytes of patients undergoing chronic haemodialysis and with kidney transplants. Clinica Chimica Acta, 115(2), 105–110. 10.1016/0009-8981(81)90065-6 7026088

[jat3930-bib-0014] Kaufmann, W. , Bolon, B. , Bradley, A. , Butt, M. , Czasch, S. , Garman, R. H. , … Sills, R. (2012). Proliferative and nonproliferative lesions of the rat and mouse central and peripheral nervous systems. Toxicologic Pathology, 40(Suppl 4), 87S–157S. 10.1177/0192623312439125 22637737

[jat3930-bib-0015] Kerr, J. , Abbenhuys, D. , & Irby, D. (1986). Crystalloid formation in Leydig cells of rats (*Rattus fuscipes*). Cell and Tissue Research, 245(1), 91–100. 10.1007/BF00218090 3731253

[jat3930-bib-0016] Kluwe, W. , Huff, J. , Matthews, H. , Irwin, R. , & Haseman, J. (1985). Comparative chronic toxicities and carcinogenic potentials of 2‐ethylhexyl‐containing compounds in rats and mice. Carcinogenesis, 6(11), 1577–1583. 10.1093/carcin/6.11.1577 4053278

[jat3930-bib-0017] Kojima, H. , Takeuchi, S. , Itoh, T. , Iida, M. , Kobayashi, S. , & Yoshida, T. (2013). In vitro endocrine disruption potential of organophosphate flame retardants via human nuclear receptors. Toxicology, 314(1), 76–83. 10.1016/j.tox.2013.09.004 24051214

[jat3930-bib-0018] Kosarac, I. , Kubwabo, C. , & Foster, W. G. (2016). Quantitative determination of nine urinary metabolites of organophosphate flame retardants using solid phase extraction and ultra performance liquid chromatography coupled to tandem mass spectrometry (UPLC‐MS/MS). Journal of Chromatography B, 1014, 24–30. 10.1016/j.jchromb.2016.01.035 26869296

[jat3930-bib-0019] Kozina, V. , Geist, D. , Kubinová, L. , Bilić, E. , Karnthaler, H. P. , Waitz, T. , … Ježek, D. (2011). Visualization of Reinke's crystals in normal and cryptorchid testis. Histochemistry and Cell Biology, 135(2), 215–228. 10.1007/s00418-011-0782-6 21287192

[jat3930-bib-0020] Li, S. , & Davis, B. (2007). Evaluating rodent vaginal and uterine histology in toxicity studies. Birth Defects Research Part B: Developmental and Reproductive Toxicology, 80(3), 246–252. 10.1002/bdrb.20120 17570136

[jat3930-bib-0021] Lobo, M. V. , Arenas, M. I. , Alonso, F. J. M. , Gomez, G. , Bazán, E. , Paíno, C. L. , … Moyano, A. (2004). Nestin, a neuroectodermal stem cell marker molecule, is expressed in Leydig cells of the human testis and in some specific cell types from human testicular tumours. Cell and Tissue Research, 316(3), 369–376. 10.1007/s00441-003-0848-4 15127288

[jat3930-bib-0022] Lubet, R. A. , Nims, R. W. , Mayer, R. T. , Cameron, J. W. , & Schechtman, L. M. (1985). Measurement of cytochrome P‐450 dependent dealkylation of alkoxyphenoxazones in hepatic S9s and hepatocyte homogenates: Effects of dicumarol. Mutation Research, 142(3), 127–131. 10.1016/0165-7992(85)90052-1 2579331

[jat3930-bib-0023] Lundgren, B. , & DePierre, J. W. (1987). Induction of xenobiotic‐metabolizing enzymes and peroxisome proliferation in rat liver caused by dietary exposure to di(2‐ethylhexyl)phosphate. Xenobiotica, 17(5), 585–593. 10.3109/00498258709043965 3111107

[jat3930-bib-0024] Lundgren, B. , Meijer, J. , Birberg, W. , Pilotti, Å. , & DePierre, J. W. (1988). Induction of cytosolic and microsomal epoxide hydrolases in mouse liver by peroxisome proliferators, with special emphasis on structural analogues of 2‐ethylhexanoic acid. Chemico‐Biological Interactions, 68(3–4), 219–240. 10.1016/0009-2797(88)90018-X 3214886

[jat3930-bib-0025] Lundgren, B. , Meijer, J. , & DePierre, J. W. (1987). Examination of the structural requirements for proliferation of peroxisomes and mitochondria in mouse liver by hypolipidemic agents, with special emphasis on structural analogues of 2‐ethylhexanoic acid. The FEBS Journal, 163(2), 423–431. 10.1111/j.1432-1033.1987.tb10815.x 3028804

[jat3930-bib-0026] MacFarland, H. , & Punte, C. Jr. (1966). Toxicological studies on tri‐(2‐ethylhexyl)‐phosphate. Archives of Environmental Health: An International Journal, 13(1), 13–20. 10.1080/00039896.1966.10664501 4957697

[jat3930-bib-0027] Mann, P. C. , Vahle, J. , Keenan, C. M. , Baker, J. F. , Bradley, A. E. , Goodman, D. G. , … Tanaka, T. (2012). International harmonization of toxicologic pathology nomenclature: An overview and review of basic principles. Toxicologic Pathology, 40(Suppl 4), 7S–13S. 10.1177/0192623312438738 22637736

[jat3930-bib-0028] Mesa, H. , Gilles, S. , Datta, M. W. , Murugan, P. , Larson, W. , Dachel, S. , & Manivel, C. (2016). Immunophenotypic differences between neoplastic and non‐neoplastic androgen‐producing cells containing and lacking reinke crystals. Virchows Archiv, 469(6), 679–686. 10.1007/s00428-016-2028-4 27696245

[jat3930-bib-0029] Mesa, H. , Gilles, S. , Smith, S. , Dachel, S. , Larson, W. , & Manivel, J. C. (2015). The mystery of the vanishing Reinke crystals. Human Pathology, 46(4), 600–606. 10.1016/j.humpath.2015.01.004 25682153

[jat3930-bib-0030] National Toxicology Program . (1984). *NTP technical report on the toxicology and carcinogenesis studies of tris(2‐ethylhexyl) phosphate (CASNO.78‐42‐2) in F344/N rats and B6C3F1 mice (gavage studies).* National Toxicology Program Technical Report Series 274. 12748680

[jat3930-bib-0031] Nolte, T. , Brander‐Weber, P. , Dangler, C. , Deschl, U. , Elwell, M. R. , Greaves, P. , … Rogers, A. (2016). Nonproliferative and proliferative lesions of the gastrointestinal tract, pancreas and salivary glands of the rat and mouse. Journal of Toxicologic Pathology, 29(Suppl 1), 1S–125S. 10.1293/tox.29.1S PMC4765498 26973378

[jat3930-bib-0032] Ono, K. , Ono, T. , & Matsumata, T. (1995). The pathogenesis of decreased aspartate aminotransferase and alanine aminotransferase activity in the plasma of hemodialysis patients: The role of vitamin B6 deficiency. Clinical Nephrology, 43(6), 405–408. 7554526

[jat3930-bib-0033] Organisation for Economic Co‐operation and Development (2008). Test No In 407: Repeated dose 28‐day Oral toxicity study in rodents. OECD: Publishing.

[jat3930-bib-0034] Padilla, S. , Lassiter, T. L. , & Hunter, D. (1999). Biochemical measurement of cholinesterase activity In T. H. A. Harry J. (Ed.), Neurodegeneration methods and protocols. Methods in molecular medicine™, vol. 22 (pp. 237–245). Totowa, NJ: Humana Press 10.1385/0-89603-612-X:237 21380839

[jat3930-bib-0035] Park, J. D. , Habeebu, S. S. , & Klaassen, C. D. (2002). Testicular toxicity of di‐(2‐ethylhexyl) phthalate in young Sprague–Dawley rats. Toxicology, 171(2), 105–115. 10.1016/S0300-483X(01)00567-4 11836017

[jat3930-bib-0036] Parmar, D. , Srivastava, S. P. , & Seth, P. K. (1986). Effect of di(2‐ethylhexyl) phthalate (DEHP) on spermatogenesis in adult rats. Toxicology, 42(1), 47–55. 10.1016/0300-483X(86)90091-0 2879365

[jat3930-bib-0037] Pass, D. , & Freeth, G. (1993). The rat. Anzccart, 6(4), 1–4.

[jat3930-bib-0038] Piché, C. D. , Sauvageau, D. , Vanlian, M. , Erythropel, H. C. , Robaire, B. , & Leask, R. L. (2012). Effects of di‐(2‐ethylhexyl) phthalate and four of its metabolites on steroidogenesis in MA‐10 cells. Ecotoxicology and Environmental Safety, 79, 108–115. 10.1016/j.ecoenv.2011.12.008 22236953

[jat3930-bib-0039] Porter, E. , Crump, D. , Egloff, C. , Chiu, S. , & Kennedy, S. W. (2014). Use of an avian hepatocyte assay and the avian toxchip polymerse chain reaction array for testing prioritization of 16 organic flame retardants. Environmental Toxicology and Chemistry, 33(3), 573–582. 10.1002/etc.2469 24273086

[jat3930-bib-0040] Reemtsma, T. , Lingott, J. , & Roegler, S. (2011). Determination of 14 monoalkyl phosphates, dialkyl phosphates and dialkyl thiophosphates by LC‐MS/MS in human urinary samples. Science of the Total Environment, 409(10), 1990–1993. 10.1016/j.scitotenv.2011.01.032 21334725

[jat3930-bib-0041] Renne, R. , Brix, A. , Harkema, J. , Herbert, R. , Kittel, B. , Lewis, D. , … Wohrmann, T. (2009). Proliferative and nonproliferative lesions of the rat and mouse respiratory tract. Toxicologic Pathology, 37(Suppl 7), 5S–73S. 10.1177/0192623309353423 20032296

[jat3930-bib-0042] Smyth, H. F. Jr. , & Carpenter, C. P. (1948). Further experience with the range finding test in the industrial toxicology laboratory. The Journal of Industrial Hygiene and Toxicology, 30(1), 63–68. 18895731

[jat3930-bib-0043] Stuer‐Lauridsen, F. , Mikkelsen, S. , Havelund, S. , Birkved, M. , Hansen, L. P. , Engineers, C. C. , & Planners, A. (2001). Environmental and health assessment of alternatives to phthalates and to flexible PVC. Environmental Project, 590.

[jat3930-bib-0044] Thoolen, B. , Maronpot, R. R. , Harada, T. , Nyska, A. , Rousseaux, C. , Nolte, T. , … Ward, J. M. (2010). Proliferative and nonproliferative lesions of the rat and mouse hepatobiliary system. Toxicologic Pathology, 38(Suppl 7), 5S–81S. 10.1177/0192623310386499 21191096

[jat3930-bib-0045] Van Esch, G. J. , & World Health Organisation (2000). Flame retardants: Tris (2‐butoxyethyl) phosphate, tris(2‐ethylhexyl) phosphate and tetrakis (hydroxymethyl) phosphonium salts. Geneva: WHO.

[jat3930-bib-0046] Verschueren, K. (2001). Handbook of environmental data on organic chemicals: Vol. 1. New York, NY: John Wiley and Sons, Inc.

[jat3930-bib-0047] Zhang, Q. , Lu, M. , Dong, X. , Wang, C. , Zhang, C. , Liu, W. , & Zhao, M. (2014). Potential estrogenic effects of phosphorus‐containing flame retardants. Environmental Science & Technology, 48(12), 6995–7001. 10.1021/es5007862 24844797

